# Inspiratory muscle fatigue at the swimming tumble turns: its occurrence and effects on kinematic parameters of the turns

**DOI:** 10.3389/fphys.2023.1219520

**Published:** 2023-06-13

**Authors:** Tadeja Moravec, Mitch Lomax, Anton Ušaj, Jernej Kapus

**Affiliations:** ^1^ Primary School Toneta Pavčka, Mirna Peč, Slovenia; ^2^ School of Sport, Health, and Exercise Science, University of Portsmouth, Portsmouth, United Kingdom; ^3^ Laboratory of Biodynamics, Faculty of Sport, University of Ljubljana, Ljubljana, Slovenia

**Keywords:** young swimmers, flip turn, fatigue, inspiratory mouth pressure, front crawl

## Abstract

**Introduction:** The present study had two objectives: 1) to investigate the effects of tumble turns on the development of inspiratory muscle fatigue (IMF) and compare this to whole swimming, and 2) to evaluate the effects of pre-induced IMF on the kinematic parameters of tumble turns. Fourteen young club-level swimmers (13 ± 2 years of ages) completed three swim trials.

**Methods:** The first trial was used to determine the 400-m front crawl swim time at maximal effort (400FC). The other two trials consisted of a series of 15 tumble turns at the 400FC pace. In one of the turn-only trials, IMF was pre-induced (TURNS-IMF), whereas in the other turn-only trial it was not (TURNS-C).

**Results:** Compared with baseline values, the values for maximal inspiratory mouth pressure (PImax) at the end of the swim were significantly lower at all trials. However, the magnitude of inspiratory muscle fatigue was less after TURNS-C (PImax decreased by 12%) than after 400FC (PImax decreased by 28%). The tumble turns were slower during 400FC than during TURNS-C and TURNS-IMF. In addition, compared to TURNS-C, turns in the TURNS-IMF were performed with higher rotation times and shorter apnea and swim-out times.

**Discussion:** The results of the present study suggest that tumble turns put a strain on the inspiratory muscles and directly contribute to the IMF observed during 400FC swimming. Furthermore, pre-induced IMF resulted in significantly shorter apneas and slower rotations during tumble turns. IMF therefore has the potential to negatively affect overall swimming performance, and strategies should be sought to reduce its effects.

## 1 Introduction

The occurrence of inspiratory muscle fatigue (IMF) has been reported after 100-m (Brown and Kilding, 2011), 200-m (Lomax and McConnell, 2003; Jakovljevic and McConnell, 2009), 300 m, and 400-m (Thomaidis et al., 2009) front crawl (FC) swimming. It has been obtained after 200-m race-paced swimming in all four strokes (Lomax et al., 2012) and at submaximal FC intensities above the critical speed (Lomax et al., 2013). The origin of IMF may lie in the breathing mechanics and the dual role of the breathing muscles during swimming. Regardless of swimming stroke, immersion increases inspiratory muscle tone (Hong et al., 1969), partially counteracts inspiratory muscle force (Hong et al., 1969; Robertson et al., 1978), and results in lung constriction (Hong et al., 1969). Consequently, both the elastic and dynamic work of breathing increase during swimming, resulting in increased work of breathing (Robertson et al., 1978). In addition, some of the trunk, chest, and dorsal muscles of the upper body used in FC, backstroke, breaststroke, and butterfly swimming are also recruited during deep inspirations (Kendall Peterson et al., 2005; McLeod, 2010), resulting in a duality of muscle function. Because these factors could decrease the functional capacity of inspiratory muscles over time, they could negatively affect swimming performance.

In all the above studies, IMF was determined after the swim test without distinguishing between the effects of the actual swim component and the turns. Research has shown the importance of turning technique to swimming performance (Chow et al., 1984; Morais et al., 2018). Specifically, during 100-m distances, turns account for 19%–20% of the total race time (Morais et al., 2018), 21% of total race time in 200-m freestyle races (Slawson et al., 2010), and 36% of total race time in 800-m freestyle races (Morais et al., 2019), performed in 50-m pools. As with swimming, turns present a challenge to the swimmer’s breathing. Swimmers perform tumble turns in FC and backstroke and open turns in butterfly and breaststroke (Slawson et al., 2010). In tumble turns, the swimmer’s head is below the surface of the water for much of the turn, which is longer than swimming alone. Swimmers should be able to take a deep breath during the last stroke cycle before the wall or even earlier. After that, they should hold their breath during the rotations around the transverse and frontal axes, the glide, and the undulating underwater swim until the swim-out. For efficient performance, they try to optimize the duration, depth and body position during the glide, start the undulating underwater swim at the right time, perform it in the optimal body position with optimal frequency and amplitude of kicking and swim-out at the right time (Ruiz-Navarro et al., 2022; West et al., 2022; Veiga et al., 2023). This could be accomplished more easily if they can take a deep breath before the tumble turn and hold it throughout the underwater post-turn phase. As with the tumble turn, swimmers should also take a deep breath during the open turn. The reason for this is that after pushing off the wall, swimmers perform a glide and swim-out with multiple kicks in the butterfly and with underwater stroke in the breaststroke. Accordingly, it is surprising that no study has yet examined the impact of turns *per se* on the development of IMF. Moreover, it is also unknown how IMF affects turn performance. Regarding general swimming performance, it is known that pre-induced IMF increases metabolic acidosis during subsequent swimming, slows swimming speed, and increases stroke rate and breathing frequency (Lomax et al., 2019). Therefore, the aim of the present study was to investigate the effects of tumble turns on the development of IMF and compare this to whole FC swimming. To do this a series of tumble turns were performed either with or without pre-induced IMF and compared to maximal 400-m FC swimming. In addition, we wanted to evaluate the effects of pre-induced IMF on the kinematic parameters of the tumble turns. We hypothesized that tumble turns by themselves would cause IMF but due to the absence of the swimming component, the magnitude of IMF would be less than after the maximal 400-m FC swim. We also hypothesized that pre-inducing IMF would increase the total turn time and the rotation time and decrease the duration of the apnea phase and the swim-out time during the tumble turns.

## 2 Materials and methods

### 2.1 Experimental approach to the problem

Swimmers completed one pulmonary familiarization and three swimming trials in an indoor heated (mean ± SD: 27.2°C ± 0.3°C) 25-m swimming pool. The first trial was used to determine maximum-effort 400-m FC swimming (400FC) time. The remaining two trials consisted of a series of 15 tumble turns at the 400FC pace. We used the series of tumble turns to avoid the fatigue arising from the actual swimming component of the 400FC. The turns consisted of a 6-m swim into the wall, turn, and a 6-m swim from the wall. The rest period between each turn reflected the elapsed time that would have occurred had the swimmer been swimming 400-m FC at race pace. The 15 tumble turns reflect the number of tumble turns occurring during a 400-m swim in a 25-m pool. We defined the turn segment as swimming from the 5-m before the wall to the 5-m after the wall push-off (Blanksby et al., 1996; Rejman and Borowska, 2008; Faelli et al., 2021). We selected, 6-m in and out of the wall so that swimmers were swimming through the 5-m measurement area at maximum speed, that is, as fast as possible past the start and finish marks. In one of the turn-only trials, IMF was pre-induced (TURNS-IMF), whereas in the other turn-only trial it was not (TURNS-C). Kinematic parameters of the turns (the total turn time, the rotation time, the apnea time and the swim-out time) were recorded during each swim and maximal inspiratory and expiratory mouth pressures (PImax and PEmax respectively) were measured before and after each experimental swim. In the case of the TURNS-IMF trial, PImax was measured before the inducement of IMF (baseline) and after the inducement of IMF (baseline after IMF inducement).

### 2.2 Participants

Fourteen young club level swimmers volunteered for this study (see Table 1 for their descriptive characteristics). Before participating in the study, the swimmers and their parents were fully informed about the aims and procedures of the study. Participants and their legal guardians gave written informed consent. All swimmers were free of cardiorespiratory diseases. Their average training experience was 5 ± 2 years. The study was conducted in accordance with the Declaration of Helsinki, and approved by the Institutional Review Board (or Ethics Committee) of National Medical Ethics Committee (protocol code 0120-425/2022/3, date of approval: 25. 11. 2022).

**TABLE 1:** Descriptive characteristic of the participants at the start of the study: mean ± SD.

Measure	Group	Males	Females
n	14	9	5
Age (years)	13 ± 2	13 ± 2	14 ± 1
Body mass (kg)	52.9 ± 14.0	51.2 ± 16.5	55.9 ± 8.6
Height (cm)	161.20 ± 15.22	159.28 ± 18.89	164.66 ± 3.95
VC (l)	3.74 ± 0.87	3.61 ± 0.94	4.02 ± 0.73
FVC (l)	3.25 ± 0.99	3.29 ± 1.12	3.17 ± 0.78
FEV_1_ (l^.^s^-1^)	2.98 ± 0.87	2.93 ± 0.97	3.08 ± 0.68
FEV1/FVC (%)	93.31 ± 8.56	91.33 ± 9.38	97.75 ± 4.5
PImax (cm $H_{2}$ O)	100 ± 25	98 ± 30	105 ± 7
PEmax (cm $H_{2}$ O)	120 ± 27	117 ± 29	124 ± 22
Time 400 FC (s)	338.7 ± 44.1	342.8 ± 44.5	331.4 ± 47.4

VC, vital capacity; FVC, forced vital capacity; FEV_1_, forced expired volume the first second of exhalation; PImax, maximal inspiratory mouth pressure; PEmax, maximal expiratory mouth pressure.

### 2.3 Pulmonary familiarisation

PImax (measured from residual volume) and PEmax (measured from total lung capacity) were determined with the nose occluded and using a hand-held respiratory pressure meter whilst standing on poolside (RPM, Micro Medical Ltd., Kent, UK). Reliability was deemed present when the three highest manoeuvres from a series of manoeuvres were within 10 cmH_2_O. Forced vital capacity (FVC) and forced expired volume in the first second of exhalation (FEV_1_) were recorded for descriptive purposes using a digital spirometer (Micro Spirometer, Micro Medical Ltd., Kent, UK). Participants completed a minimum of three technically satisfactory manoeuvres with the highest recorded.

### 2.4 Swimming trials

The participants completed three swimming trials. All of them were initiated from a push start, completed using the FC stroke, and occurred on separate days. The 400FC trial was started by pushing off the wall. In the sets of 15 turns, the swimmers started with the push-off from the bottom of the pool. An 800-m warm-up consisting of all swimming strokes was completed before each trial. The first trial was the 400FC. The remaining two trials, TURNS-C and TURNS-IMF, were administered in a counterbalanced order. During these trials participants were instructed to swim in and out of the turn at maximum speed from and to the 6-m mark. The appropriate rest period was given as described earlier. A researcher informed participants of the total time achieved for each turn and monitored the duration of rest during the series of tumble turns.

### 2.5 Inspiratory muscle fatigue inducement

A commercially available inspiratory muscle trainer (POWERbreathe, H&B International, Southam, Warwickshire, United Kingdom) was used to induce IMF before the series of tumble turns in the TURNS-IMF trial. While standing and with the nose occluded, participants breathed through a flanged rubber mouthpiece connected to the inspiratory muscle trainer. The 1-way inspiratory value inside the trainer was set to open when participants produced 70% of their predetermined PImax (expiratory muscles were not loaded). A duty cycle of 0.60 was adopted (3 s for inspiration, 2 s for expiration) and an $f_{b}$ (breaths per minute) of 12 throughout the loading regime (Gallagher et al., 1985). Participants coordinated inspiration and expiration using a metronome and maintained this pattern until it could no longer be sustained for 3 consecutive breaths (Lomax and Castle, 2011). Participants then continued for a further minute after which PImax was re-assessed and recorded as baseline after IMF inducement. This loading regime has been shown to result in a fall in PImax of 17%–25% in swimmers (Lomax and Castle, 2011; Lomax et al., 2014), which is consistent with the magnitude (8%–29%) observed following 100-m and 400-m FC swimming (Lomax and McConnell, 2003; Jakovljevic and McConnell, 2009; Thomaidis et al., 2009; Brown and Kilding, 2011; Lomax et al., 2013). Due to hyperventilation arising from the increase in breathing depth, mild hypocapnia and alkalosis may develop as result of the IMF protocol (Lomax et al., 2019). Results of previous studies show that about 1–2 min are required to return breathing pattern and Pco_2_ to resting values after the cessation of hyperventilation (Tawadrous and Eldridge, 1974; Meah and Gardner, 1994). Therefore, once IMF was confirmed by the fall in PImax at baseline after IMF inducement, swimmers waited 5 min before commencing the series of tumble turns.

### 2.6 Video analysis setting and measurements

A 2D video analysis was performed using Kinovea software 0.8.15 (Copyright ^©^ 2006–2011, Joan Charmant & Contrib). Each trial was recorded by two cameras (one under water, the other above water). The first underwater camera (GoPro^®^ HERO5, 60Hz at 120 fps and with a resolution of 720 pixel) was attached to a trolley at a depth of 0.36 m. The trolley was pulled by the operator at the same speed as the swimmer, with the head of the participant being the marker that the operator followed to control parallax (Seifert et al., 2005). The second camera was positioned above the lateral wall of the pool, on a ladder situated at a distance 5-m from the turning wall and at a height of 1.7-m from the floor. A black rubber band on the rope in the pool lane, 5-m away from the turning wall served as a reference point for the video analysis of the selected kinematic variables. It should be noted that available race analysis studies are not consistent in their analytical methods. For example, some studies have defined the turn segment as from the 5-m before the wall until the 15-m after the wall push-off (Morais et al., 2018; Marinho et al., 2020), while other studies have considered the same segment as 7.5-m before and after the wall (Veiga et al., 2013). We took the total turn duration over 5-m as an indicator of turn performance and using a longer duration or distance would have included more swimming (Blanksby et al., 1996).

In each trial, 15 turns were analysed using Kinovea software. We measured the time durations of: 1) the turn, which started with the frame when the swimmer’s head reached the 5-m mark towards the turn wall and ended with the frame when the swimmer’s head reached the 5-m mark again after the turn; 2) the rotation, which started with the frame when the head and body started to lean down and ended with the frame when the feet touched the wall; 3) the apnea, which started with the frame that corresponded to the last breath before the turn and ended with the frame that corresponded to the first breath after it; and 4) the swim-out, which started with the frame when the toes were pulled back from the wall and ended with the frame when the ear broke the water surface. Two researchers performed video analysis to identify the initial and final frames of each analyzed phase. We used phase identification similar to that used in previous studies (Lyttle and Mason, 1997; Puel et al., 2012; Skyriene et al., 2017; Veiga and Roig, 2017; Morais et al., 2018; 2019; Marinho et al., 2020; Faelli et al., 2021).

PImax and PEmax were recorded pre- (baseline) and post- (end-swim) the 400FC and TURNS-C trials using a hand-held respiratory pressure meter (Micro RPM, MicroMedical Ltd., Kent, United Kingdom). The values were used to assess IMF and expiratory muscle fatigue (EMF), respectively. In the case of the TURNS-IMF trial, baseline consisted of a pre-IMF inducement value (baseline) and a post-IMF inducement value (baseline after IMF inducement). Manoeuvres were made from a standing position on poolside (with the nose occluded) before and within 60 s following each trial. PImax was recorded from residual volume whereby participants were instructed to fully empty their lungs then inhale as hard and as fast as possible for approximately two to three s. PEmax was measured from total lung capacity and participants were instructed to fill up their lungs and exhale as hard and as fast as possible for two to three s. Before (baseline) and 1 minute after the end of each trial, blood samples were taken from the finger capillary to determine lactate ([ ${LA}^{-}$ ]) (Lactate Pro™ (Arkray KDK, Japan)). Additionally, heart rate (HR) was recorded at the beginning and at the end of all trials (Polar OH1+).

### 2.7 Statistical analyses

The sample size was calculated considering a two-way repeated measures ANOVA with three interventions (400FC, TURN-C and TURN-IMF) and two repeated measurements (pre- and post-swim). The main endpoint was the PImax. This is the first study to examine the occurrence of a PImax decrease in response to a set of tumble turns. Therefore, our sample size calculation could only be based on previous studies of IMF in swimming. Swimming at 200-m race pace FC caused a significant PImax decrease (*p* < 0.05) with an effect size of 0.72 (Lomax et al., 2012). In the preliminary experiment we obtained a correlation of PImax values measured before and after a set of tumble turns in the 400FC of 0.82 (Pearson’s correlation coefficient; *p* < 0.05). The nonsphericity correction was defined as 1.0. With these parameters, we needed 9 participants [which was less than the actual sample size (*n* = 14)] to find a *p*-value of less than 0.05, assuming a power of 0.80 (G*Power 3.1, University of Düsseldorf, Germany).

The magnitude of IMF and EMF were defined by the transient reduction in mouth pressure pre- (baseline PImax and PEmax) vs. end-swim, expressed as a percentage of the pre-test value. The turn parameters were averaged for the first seven turns and for the last seven turns and then used for the further analyses. This also permitted the change (∆) from the first to the last seven turns to be calculated.

All statistical analyses were carried out with SPSS (IBM SPSS Statistics 21.0.). Normality of the distribution was assessed with the Shapiro-Wilk test, and sphericity was tested with the Mauchly test. A two-way analysis of variance (ANOVA) was performed using trials (400FC, TURN-C, and TURN-IMF) and time (pre-vs. post-trial or the first vs last seven turns) as factors and outcome measures (PImax, PEmax, HR [LA^−^], the turn parameters) as dependent variables. A one-way analysis of variance (ANOVA) was used to test for differences in IMF (as a calculated variable) between trials. When a significant interaction was detected (the F-ratio was significant), pairwise comparisons were performed with LSD (confidence interval adjustment). Moreover, the observed power and the effect size was calculated to estimate the variance between trials [partial eta squared: (η2)] and Cohen’s *d* for pairwise comparisons. The following guidelines were used to interpret the strength of η2 values (Cohen, 1988): 0.01 = small effect; 0.06 = moderate effect; and 0.14 = large effect. A Cohen’s *d* of 0.2 was deemed small, 0.6 moderate, 1.2 large, 2.0 very large and 4.0 extremely large (Hopkins et al., 2009). Pearson’s correlation coefficient was used to determine any relationships among the variables obtained. Significance was set *a priori* as *p* < 0.05. Data are presented as mean ± SD.

## 3 Results

**FIGURE 1:**
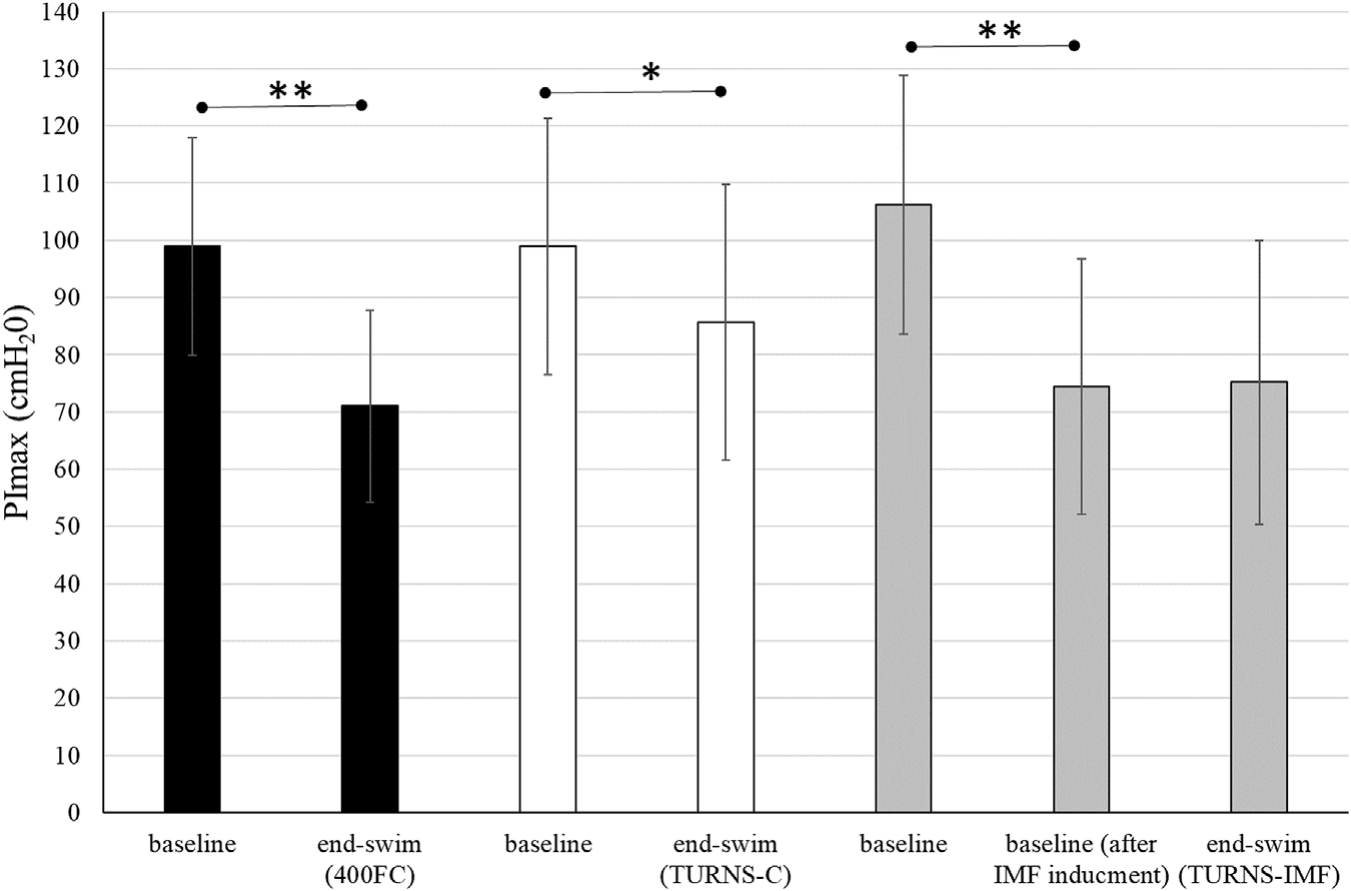
PImax immediately before the swim and end-swim in the 400FC (black bars), the TURNS-C (white bars) and the TURNS-IMF (grey bars). Two-way ANOVA followed by the LSD (confidence interval adjustment), * indicates significant difference between the measurements within trial (*p* < 0.05); ** indicates significant difference between the measurements within trial (*p* < 0.01).

All participants successfully completed all three trials. They finished tumble turns and swam-out before the 5-m mark. Baseline PImax was similar in all trials (Figure 1). In the TURNS-IMF trial, pre-inducing IMF (baseline after IMF inducement in Figure 1) caused PImax to fall by 32 ± 10 cmH_2_O from 106 ± 23 cmH_2_O (F = 131.50, *p* < 0.01, *d* = 3.2, observed power = 1, 95% confidence interval 26–38), but no further reduction occurred in response to a series of 15 tumble turns (F = 0.03, *p* > 0.05, *d* = 0.05, observed power = 0.05, mean difference 0.8 cmH_2_O; 95% confidence interval -10–11). In comparison to baseline values (before IMF inducement for TURNS-IMF), PImax values at the end of swimming were significantly lower in all three trials (F = 92.73, *p* < 0.01, observed power = 1, mean difference 28 cmH_2_O; 95% confidence interval 22-34 in the 400FC, F = 8.4, *p* < 0.05, observed power = 0.76, mean difference 13 cmH_2_O; 95% confidence interval 3-23 in the TURNS-C and F = 45.34, *p* < 0.01, observed power = 1, mean difference 31 cmH_2_O; 95% confidence interval 21-41 in the TURNS-IMF). Large effect sizes were observed in all three trials (*d* = 2.4 in the 400FC; *d* = 0.8 in the TURN -C; *d* = 1.7 in the TURN-IMF). There was no significant difference in end-swim PImax between trials (F = 2.65, *p* > 0.05, η2 = 0.31, observed power = 0.43).

**FIGURE 2:**
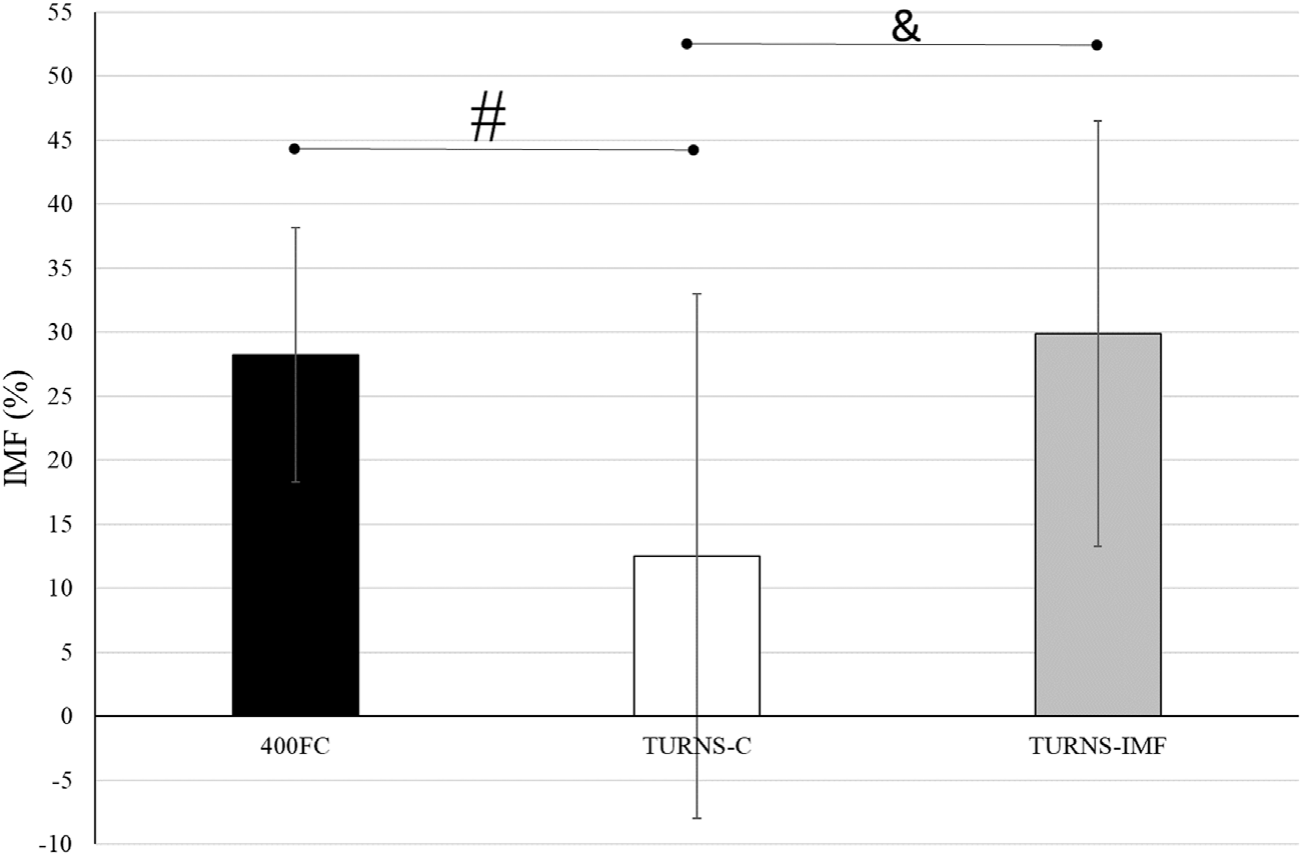
A comparison of IMF after swim trials between 400FC (black bars), TURNS-C (white bars) and TURNS-IMF (grey bars). One-way ANOVA followed by the LSD (confidence interval adjustment), # indicates significant difference between 400FC and TURNS-C (*p* ≤ 0.05); & indicates significant difference between TURNS-C and TURNS-IMF (*p* ≤ 0.05).

There were significant differences in IMF between swim trials (F = 5.60, *p* < 0.01, η2 = 0.30, observed power = 0.81) (Figure 2). The magnitude of IMF was less following TURNS-C (12% ± 20%) in comparison to 400FC (28% ± 10%) (F = 6.67, *p* = 0.02, *d* = 0.7, observed power = 0.67, mean difference 16%; 95% confidence interval 3–29) and to TURNS-IMF (29% ± 17%) (F = 7.65, *p* = 0.02, *d* = 0.7, observed power = 0.73, mean difference 17%; 95% confidence interval 4–31).

We measured PEmax only in the TURN-C and TURN-IMF. There was no significant difference between the trials in the baseline values of this parameter (111 ± 24 cmH_2_O in the TURN-C and 114 ± 25 cmH_2_O in the TURN-IMF; F = 1.71, *p* = 0.21, *d* = 0.4, observed power = 0.23). End-swim PEmax decreased to 91 ± 23 cmH_2_O only in the TURN-IMF (F = 15.64, *p* = 0.00, *d* = 1.1, observed power = 0.95, mean difference 23 cmH_2_O; 95% confidence interval 10–35). End-swim PEmax was lower (F = 4.60, *p* = 0.05, *d* = 0.6, observed power = 0.51, mean difference 14 cmH_2_O; 95% confidence interval −0.1–28) in the TURNS-IMF trial (91 ± 23 cmH_2_O) compared to the TURNS-C trial (105 ± 18 cmH_2_O).

Table 2 shows that tumble turns slowed down during the trials (F = 40.52, *p* < 0.01, η2 = 0.76, observed power = 1). In addition, the turns were slowest during 400FC (F = 73.88, *p* < 0.01, η2 = 0.85, observed power = 1). The turn durations in this trial were significantly longer compared to TURNS-C (mean difference 0.96 s; 95% confidence interval 0.75–1.16, *p* < 0.01, *d* = 2.8 in the first seven turns; mean difference 1.21 s; 95% confidence interval 0.94–1.49, *p* < 0.01, *d* = 2.6 in the last seven turns) and TURNS-IMF (mean difference 0.85 s; 95% confidence interval 0.67–1.03, *p* < 0.01, *d* = 2.8 in the first seven turns; mean difference 1.08 s; 95% confidence interval 0.87–1.30, *p* < 0.01, *d* = 2.8 in the last seven turns). However, there were no significant differences in total time durations between TURNS-C and TURNS-IMF.

**TABLE 2:** Turn parameters obtained during the maximal 400 FC swim (400FC), and during the series of 15 tumble turns without pre-induced IMF (TURNS-C) and with it (TURNS-IMF).

Parameter turns		400FC	TURNS-C		TURNS-IMF		
Total turn time (s)	First seven	7.72 ± 1.08	6.76 ± 0.97	##	6.86 ± 1.01		✓✓
	Last seven	8.05 ± 1.12**	6.83 ± 0.99	##	6.96 ± 1.03**		✓✓
Apnea time (s)	First seven	4.23 ± 0.48	5.03 ± 0.67	##	4.24 ± 0.59	&&	
	Last seven	3.92 ± 0.44**	4.53 ± 0.70**	##	3.95 ± 0.55**	&&	
Rotation time (s)	First seven	1.00 ± 0.09	0.81 ± 0.07	##	0.86 ± 0.09	&&	✓✓
	Last seven	1.05 ± 0.09**	0.81 ± 0.08	##	0.85 ± 0.09	&&	✓✓
Swim-out time (s)	First seven	1.68 ± 0.45	1.98 ± 0.56	##	1.70 ± 0.54	&&	
	Last seven	1.43 ± 0.32**	1.79 ± 0.56**	##	1.59 ± 0.57*	&	

Data are presented as means ± standard deviations for each trial. Two-way ANOVA followed by the LSD (confidence interval adjustment), * indicates significant difference between the first and the last seven turns in a particular trial (*p* ≤ 0.05); ** indicates significant difference between the first and the last seven turns in a particular trial (*p* ≤ 0.01); ## indicates significant difference between 400FC and TURNS-C (*p* ≤ 0.01); & indicates significant difference between TURNS-C and TURNS-IMF (*p* ≤ 0.05); && indicates significant difference between TURNS-C and TURNS-IMF (*p* ≤ 0.01); ✓✓ indicates significant difference between 400FC and TURNS-IMF (*p* ≤ 0.01).

Apnea duration during tumble turns shortened throughout the trials (F = 76.43, *p* < 0.01, η2 = 0.86. observed power = 1) (Table 2). In addition, there were significant differences in apnea times between the trials (F = 19.22, *p* < 0.01; η2 = 0.6, observed power = 1). Apnea times were higher in the TURNS-C than in the 400FC (mean difference 0.8 s; 95% confidence interval 0.53–1.08, *p* < 0.01, *d* = 1.7 in the first seven turns; mean difference 0.61 s; 95% confidence interval 0.25–0.96, *p* < 0.01, *d* = 1.0 in the last seven turns) and in the TURNS-IMF (mean difference 0.79 s; 95% confidence interval 0.53–1.05, *p* < 0.01, *d* = 1.8 in the first seven turns; mean difference 0.58 s; 95% confidence interval 0.26–0.90, *p* < 0.01, *d* = 1 in the last seven turns). However, there were no significant differences in apnea durations between 400FC and TURNS-IMF (*p* > 0.05).

The factor time interacted significantly with rotation time during tumble turns (F = 8.74, *p* = 0.01, η2 = 0.40, observed power = 0.78) (Table 2). Rotation slowed during the 400FC (mean difference 0.05 s; 95% confidence interval 0.03–0.07, *p* < 0.01, *d* = 1.5), but not during the TURNS-C and TURNS-IMF. Moreover, there were significant differences in the duration of rotation between trials (F = 151.77, *p* < 0.01; η2 = 0.92, observed power = 1). Participants made the faster rotation during the TURNS-C compared to the TURNS-IMF (mean difference 0.05 s; 95% confidence interval 0.02–0.08, *p* < 0.01, *d* = 1.1 in the first seven turns; mean difference 0.04 s; 95% confidence interval 0.02–0.06, *p* < 0.01, *d* = 1.1 in the last seven turns) and the 400FC (mean difference 0.19 s; 95% confidence interval 0.15–0.24, *p* < 0.01, *d* = 2.6 in the first seven turns; mean difference 0.25 s; 95% confidence interval 0.21–0.28, *p* < 0.01, *d* = 3.8 in the last seven turns). In addition, rotation times were significantly longer in the 400FC than in the TURNS-IMF (mean difference 0.14 s; 95% confidence interval 0.11–0.17, *p* < 0.01, *d* = 2.6 in the first seven turns; mean difference 0.2 s; 95% confidence interval 0.17–0.24, *p* < 0.01, *d* = 3.4 in the last seven turns).

Swim-out time during tumble turns decreased throughout all trails (F = 41.59, *p* < 0.01; η2 = 0.76, observed power = 1) (Table 2). In addition, there were significant differences in the duration of the swim-out between trials (F = 9.05, *p* < 0.01; η2 = 0.41, observed power = 0.96). Swim-out times were higher in the TURNS-C (mean difference 0.3 s; 95% confidence interval 0.11–0.49, *p* < 0.01, *d* = 0.9 in the first seven turns; mean difference 0.35 s; 95% confidence interval 0.13–0.58, *p* < 0.01, *d* = 0.9 in the last seven turns) than in the 400FC and TURNS-IMF (mean difference 0.28 s; 95% confidence interval 0.12–0.44, *p* < 0.01, *d* = 1.0 in the first seven turns; mean difference 0.20 s; 95% confidence interval 0.03–0.37, *p* < 0.05, *d* = 0.7 in the last seven turns).

End-swim [ ${LA}^{-}$ ] was significantly higher than baseline in all three swim trials. Values increased from 2.4 ± 0.8 to 8.1 ± 3.1 mmol/l in the 400FC (F = 43.19, *p* < 0.01, *d* = 1.8, observed power = 1, mean difference 5.7 mmol/l; 95% confidence interval 3.9–7.6), from 2.8 ± 1.2 to 4.7 ± 2.2 mmol/l in the TURNS-C (F = 11.25, *p* < 0.01, *d* = 0.9, observed power = 0.87, mean difference 1.9 mmol/l; 95% confidence interval 0.7–3.0), and from 2.7 ± 1.0 to 4.7 ± 2.6 mmol/l in the TURNS-IMF (F = 7.96, *p* < 0.05, *d* = 0.8, observed power = 0.74, mean difference 2.0 mmol/l; 95% confidence interval 0.5–3.5). There was significant difference between the trials in end-swim $\left[{LA}^{-}\right]$ (F = 15.99, *p* < 0.01, η2 = 0.73, observed power = 0.99). This significant difference persisted between the 400FC and the TURNS-C (*p* = 0.000, *d* = 1.4, mean difference 3.4 mmol/l; 95% confidence interval 2.0–4.9) and between 400FC and TURNS-IMF (*p* = 0.000, *d* = 1.5, mean difference 3.5 mmol/l; 95% confidence interval 2.2–4.8). Heart rate also increased significantly during the 400FC, the TURNS-C, and the TURNS-IMF compared to baseline (in the 400FC from 85 ± 6 to 182 ± 20; F = 296.64, *p* < 0.01, *d* = 4.6, observed power = 1, mean difference 97; 95% confidence interval 85-109; in the TURNS-C from 91 ± 10 to 158 ± 34; F = 47.97, *p* < 0.01, *d* = 1.8, observed power = 1, mean difference 68; 95% confidence interval 46-89; and in the TURNS-IMF from 85 ± 16 to 159 ± 29; F = 79.65, *p* < 0.01, *d* = 2.3, observed power = 1, mean difference 75; 95% confidence interval 57–93). Heart rates at the end of swim were not significantly different between trials (F = 3.43, *p* > 0.05, η2 = 0.36, observed power = 0.53).

No correlations were observed between the magnitude of IMF and turn parameters in the 400FC trial. However, end-swim PImax correlated strongly with the total turn time (r was −0.77 and −0.76 for the first and last seven turns, respectively, *p* = 0.01). PImax also correlated moderately with the rotation time (r was −0.56 and −0.66 for the first and last seven turns, respectively, *p* ≤ 0.05), the swim-out time (r was 0.54 and 0.62 for the first and for the last seven turns, respectively, *p* ≤ 0.05) and end-swim [LA^−^] (r = 0.56, *p* = 0.04). In the TURNS-C trial, the magnitude of IMF correlated moderately with ∆ apnea time (r = 0.59, *p* = 0.03), ∆ rotation time (r = 0.65, *p* = 0.01), and ∆ HR (r = −0.57, *p* = 0.03). Moreover, end-swim PImax correlated moderately with the rotation time (r = −0.57, *p* = 0.03 for the last seven turns) and the swim-out time (r was 0.6, *p* = 0.02 for the first seven turns and r = 0.57, *p* = 0.03 for the last seven turns). In the TURNS-IMF trial, end-swim PImax was moderately correlated with the swim-out (r = 0.57, *p* = 0.03) and rotation (r = −0.64, *p* = 0.01) times of the last seven turns. The magnitude of EMF was correlated strongly with Δ in rotation times during the TURNS-IMF (r = 0.72, *p* = 0.04). End-swim PEmax was correlated with apnea times of the first seven turns (r = 0.56, *p* = 0.04) and with the rotation times (r = −0.67, *p* = 0.01) of the last seven turns.

## 4 Discussion

To our knowledge, this is the first study to determine whether IMF occurred in response to a series of tumble turns and to investigate the effects of IMF on tumble turn kinematic parameters. The main findings were: 1) IMF occurred in response to tumble turns *per se*; 2) the magnitude of IMF was less after a series of 15 tumble turns than after maximal 400-m FC swimming; and 3) pre-induced IMF decreased both apnea and swim-out times and increased rotation time during a series of tumble turns, but total turn duration was unaffected.

The magnitude of IMF observed after 400FC (28%; Figure 1) was consistent with the 8%–29% observed across different swimming strokes (Lomax et al., 2012) and FC distances ranging from 100-m to 400-m (Lomax and McConnell, 2003; Thomaidis et al., 2009; Brown and Kilding, 2011). The functional weakening of inspiratory muscles after maximal 400-m FC swimming is consistent with an increase in inspiratory muscle work caused by: a) immersion and a horizontal body position; b) stroke-induced restricted breathing; and c) propulsion demands. Immersion increases the hydrostatic pressure around the chest, which pushes the chest wall inward when the inspiratory muscles are relaxed (Withers and Hamdorf, 1989; Frangolias and Rhodes, 1996). Because hydrostatic pressure opposes the force of the inspiratory muscles and deforms the chest wall (Frangolias and Rhodes, 1996), it is believed that the work performed by the inspiratory muscles must be increased to overcome these factors (Lomax and McConnell, 2003). In addition, the ability of the inspiratory muscles to generate force is limited, being approximately 16% less in the supine position than in the upright position (Lomax and McConnell, 2003). Moreover, immersion forces the swimmer to coordinate breathing and stroke technique. During swimming, respiratory frequency tends to be lower and tidal volume higher than during spontaneous breathing (Dicker et al., 1980; Rodríguez, 2000) with tidal volume increasing in proportion to the breathing frequency restriction (Town and Vanness, 1990). In addition, a mechanically efficient stroke technique requires that inspiration time be minimized. Thus, to maintain minute ventilation at a level that meets metabolic demands, inspiration must be deep and rapid. Consequently, inspiratory muscles must produce a greater shortening velocity over a greater range of lung volumes and therefore operate at mechanically unfavourable portions of their length-tension and force-velocity relationships (Ameredes and Clanton, 1990; Aliverti et al., 1997). Furthermore, in swimming, the breath is exhaled during the recovery phase of the swim stroke while the face is submerged. Therefore, the expiratory muscles are of particular importance given that they are used during exhalation in water, i.e., against a higher pressure than in air during activities on land. Last but not least, some respiratory muscles are also involved in the generation of propulsion. Due to the duality of muscle function in FC swimming, muscles such as the latissimus dorsi, pectoralis major, and serratus anterior contribute to the increased demands of breathing as well as propulsion and stabilization (Lomax et al., 2014).

Beside IMF after the maximal 400-m FC swim, this is the first time IMF has been demonstrated in response to tumble turns specifically. As expected, the PImax drop after the TURNS-C was smaller (PImax decreased by 12%) than after the 400FC (PImax decreased by 28%) (Figure 2). As both trials were similar in terms of duration and intensity, we attribute this finding to the omission of the swimming component in the TURNS-C trial. Therefore, this is the first study to show that tumble turns can elicit IMF. The origin of the IMF after the TURNS-C could reflect the unique breathing pattern that occurs in the lead-up to the tumble turn and the turn itself. Although we did not measure tidal volume or inhalation time, it is likely that participants inhaled more deeply before the tumble turns compared with breathing during the swim as this would provide sufficient ventilation for the additional breath-hold period. During the tumble turns, the swimmer’s head is below the surface of the water during most of the turn and it has been suggested that swimmers must be able to hold their breath for at least 5 s to execute the tumble turn and resultant underwater kicking phase (Grossman et al., 2021). Indeed, apnea time in the present study ranged from 3.9 to 5.1 s (from 55% to 63% of the total turn time) and from 6.5 to 6.8 s (98% of the total turn time), respectively, for the turns during 400FC and TURNS-C, which are consistent with times obtained during 500-yard swimming competitions (Craig, 1986). During breath-holding there is progressive pressure development against the glottis, resulting in increased muscular recruitment of both inspiratory and expiratory muscles (Burtch et al., 2017). This leads to an increase in elastic breathing load as lung compliance decreases with increasing lung volume. In addition, there is a functional weakening of the inspiratory muscles as lung volume increases, due to their length-tension relationship (Rahn et al., 1946). This could lead to an increase in the relative intensity of inspiratory muscle work during tumble turns and consequently the IMF observed in the current study during the TURNS-C trial. This finding agrees with Jakovljevic and McConnell (2009) who observed a greater magnitude of IMF when breathing frequency was restricted compared with the usual FC breathing pattern. Indeed, when breathing frequency is reduced prolonged apnea occurs between inspiration and expiration and subjects increase their tidal volume in an attempt to maintain minute ventilation (Town and Vanness, 1990). Thus, we suggest that the greater magnitude of IMF observed following the 400FC trial reflects a combination of the prolonged breath hold during tumble turns and additional fatigue arising from swimming locomotion itself. Indeed, the observation that tumble turn durations were shorter during the TURN-C than 400FC trial (Table 2) may simply reflect reduced levels of swim-induced fatigue in the TURNS-C. The nature of this trial meant that swimmers received additional rest between tumble turns and engaged in less swimming locomotion compared with the 400FC trial. It is therefore not surprising that negative correlations (r = −0.77 and −0.76) were observed between PImax and the total turn times during the 400FC trial.

When IMF was pre-induced prior to the series of tumble turns, swimmers began the trial with significant IMF. Interestingly, PImax did not fall further over the course of the 15 tumble turns in the TURNS-IMF trial (Figure 1). Consequently, the overall magnitude of IMF following both 400FC and TURNS-IMF was similar (Figure 2), which is consistent with past studies (Lomax et al., 2014; Lomax et al., 2019). Nevertheless, pre-inducing IMF prolonged rotation times and shortened both apnea and swim-out times in comparison to TURNS-C condition (Table 2). We obtained relationships between the magnitude of IMF or end-swim PImax and apnea-related turn parameters (apena time and swim-out time) in the TURN-C and TURN-IMF. The IMF magnitude correlated with the ∆ in the apnea time during TURNS-C (r = 0.59). That is, the greater the IMF, the shorter apnea became throughout the series of tumble turns. In addition, PImax at the end all three trials correlated positively with the swim-out time (r = 0.54 and 0.62 in the 400FC; r = 0.60 and 0.57 in the TURNS-C, r = 0.57 in the TURNS-IMF). This suggests that transient weakening of the inspiratory muscles, whatever the cause (e.g., breath-hold, swimming-induced), contributes to shortened swim-out times as swimmers are forced to surface earlier, probably to relieve dyspnea, which is known to intensify in the presence of IMF (Mador and Acevedo, 1991). Alternatively, a higher Pco_2_ secondary to restricted breathing and maximal swimming could be another reason (Craig, 1986) especially in the 400FC trial. However, we think it likely that the rest periods permitted between tumble turns in the TURNS-C and TURNS-IMF would have allowed swimmers to exhale a higher Pco_2_ making this possibility unlikely in these trials. It should be emphasized that a longer apnea time does not automatically equate to a faster turn. The apnea period during a tumble turn begins with the last breath before the turn and ends with the first breath after the turn. Faelli et al. (2021) showed that not breathing during the last stroke into the wall allowed swimmers to maintain speed while approaching the wall, to start the rotation farther from the wall, and to rotate faster. Velocity is greater after pushing from the wall compared with swimming speed and so when velocity decreases to swimming speed, swimming is resumed (Ruiz-Navarro et al., 2022; West et al., 2022; Veiga et al., 2023). A good post-turn underwater phase begins with an effective push off from the wall followed by the maintenance of good streamlining while gliding. An effective underwater kick and swim-out must also be initiated at the right time (Mason and Cossor, 2001). In light of this, longer apnea does not automatically mean a faster tumble turn. However, the ability to hold the breath longer allows the swimmer to execute the tumble turn more effectively and choose the best time to resume swimming. This is especially important for young, less experienced swimmers such as the participants in the present study. Shortening apnea time is often not beneficial for their turn performance and usually means that they simply lifted their head before the turn or took a breath into the turn and/or swam-out earlier because they cannot hold their breath (Wen et al., 2016).

As already stated, the rotation times during the TURNS-IMF trial were longer than those during the TURNS-C (Table 2), varying from 0.79 to 0.83 s in the TURNS-C and from 0.82 to 0.90 s in the TURNS-IMF (during the 400FC trial they varied from 0.93 to 1.08 s), which is consistent with the results of (Skyriene et al., 2017). Moreover, the magnitude of the IMF correlated with the Δ (increase) of the rotation time during TURNS-C (r = 0.65), as did PImax at the end of the TURNS-C (r = −0.52 and −0.57) and TURNS-IMF (r = −0.64) trials (as well as the 400FC trial: r = −0.56 and −0.66). We should emphasize that only in the TURN-C trial was the IMF allowed to develop naturally over the course of the turns, whereas in the TURNS-IMF trial it was already present at the start and remained at that level throughout - hence no correlation was observed between IMF magnitude and rotation time. This suggests that the transient weakening of the inspiratory muscles contributes to the longer rotation times. Interestingly, rotation time during the last seven turns of the TURNS-IMF trial was also correlated with end-swim PEmax (r = −0.67, *p* = 0.01), as was the magnitude of EMF (r = 0.72, *p* = 0.00). This is not surprising given the requirement to flex the trunk during the rotation phase of the tumble turn, which is initiated by the upper fibres of the rectus abdominis and then maintained by the lower fibres of the rectus abdominis, with both obliques providing support (McLeod, 2010). Together with the internal intercostal muscle and the transverse abdominal muscle, the rectus abdominis and internal and external obliques also assist active expiration (Ratnovsky et al., 2008). It therefore follows that if these muscles experience fatigue, rotation time could be negatively affected. As we did not measure PEmax after we induced IMF at baseline, we are unable to verify whether EMF occurred in response to the series of tumble turns *per se* or whether it was an un-intended consequence of inducing-IMF. For example, it has been shown previously that pre-inducing IMF with an identical IMF regime may reduce PEmax by 15% ± 11% (Lomax et al., 2014). Despite no load being presented to the expiratory muscles during the IMF inducement regime, expiratory muscles are likely recruited to support the increased ventilatory demand (Laghi et al., 1998) and hence EMF may occur. As we did not observe a fall in end-swim PEmax, nor observe significant correlations between rotation time and either end-swim PEmax or EMF magnitude in the TURNS-C trial (i.e., in the trial without artificially induced IMF), it seems likely that the EMF observed in the TURNS-IMF trial was an un-intended consequence of pre-inducing IMF. Regardless of the cause of EMF observed in the present study, slower rotation times are not beneficial for tumble turn performance. In support of this (Skyriene et al., 2017), the positive correlations observed between the rotation time and total turn time, suggest that a slower rotation usually means a slower turn.

### 4.1 Limitations and further research

In retrospect, not measuring gliding distance and initial rolling distance was a missed opportunity as this could have been compared to data from previous studies of kinematic analysis of tumble turns (Pereira et al., 2015; Nicol et al., 2021). Future researcher should readdress this omission and verify whether IMF shortens both gliding duration and gliding distance after the tumble turn. Moreover, we did not measure PEmax after 400FC and after IMF inducement. Due to the fact that expiratory muscles are involved in the rotation phase of the tumble turn (McLeod, 2010), future investigations should include measures of expiratory muscle function. In addition, larger and more diverse samples should be selected to help generalize the findings to other populations.

## 5 Conclusion

In conclusion, the results of the present study suggest that tumble turns put a strain on the inspiratory muscles and directly contribute to the IMF observed during FC swimming. Unsurprisingly, the magnitude of IMF during the turns only trial was much lower than that observed during the maximal 400-m FC swim, whereby the overall activity of the dual role breathing and propulsion muscles will be greater. Moreover, pre-induced IMF resulted in significantly shorter apneas and slower rotations during tumble turns. A greater ability to hold the breath (that is a longer apnea time at the tumble turn), allows the swimmer to recognize the best moment to swim-out and to resume swimming with greater ease. Moreover, given that faster rotation is associated with better tumble turns, IMF therefore has the potential to negatively affect overall swimming performance, and strategies should be sought to reduce its impact.

## Data Availability

The raw data supporting the conclusions of this article will be made available by the authors, without undue reservation.
